# Biomass Accumulation, Photosynthetic Traits and Root Development of Cotton as Affected by Irrigation and Nitrogen-Fertilization

**DOI:** 10.3389/fpls.2018.00173

**Published:** 2018-02-15

**Authors:** Zongkui Chen, Xianping Tao, Aziz Khan, Daniel K. Y. Tan, Honghai Luo

**Affiliations:** ^1^Key Laboratory of Oasis Eco-Agriculture, Xinjiang Production and Construction Group, College of Agriculture, Shihezi University, Shihezi, China; ^2^Center of Agricultural Technique Extension of Manasi County, Manasi, China; ^3^Key Laboratory of Plant Genetics and Breeding, College of Agriculture, Guangxi University, Nanning, China; ^4^Faculty of Science, Plant Breeding Institute, Sydney Institute of Agriculture, School of Life and Environmental Sciences, The University of Sydney, Sydney, NSW, Australia

**Keywords:** cotton, fertigation, biomass, antioxidants, photosynthesis, root growth

## Abstract

Limitations of soil water and nitrogen (N) are factors which cause a substantial reduction in cotton (*Gossypium hirsutum* L.) yield, especially in an arid environment. Suitable management decisions like irrigation method and nitrogen fertilization are the key yield improvement technologies in cotton production systems. Therefore, we hypothesized that optimal water-N supply can increase cotton plant biomass accumulation by maintaining leaf photosynthetic capacity and improving root growth. An outdoor polyvinyl chloride (PVC) tube study was conducted to investigate the effects of two water-N application depths, i.e., 20 cm (H_20_) or 40 cm (H_40_) from soil surface and four water-N combinations [deficit irrigation (W_55_) and no N (N_0_) (W_55_N_0_), W_55_ and moderate N (N_1_) (W_55_N_1_), moderate irrigation (W_75_) and N_0_ (W_75_N_0_), W_75_N_1_] on the roots growth, leaf photosynthetic traits and dry mass accumulation of cotton crops. H_20_W_55_N_1_ combination increased total dry mass production by 29–82% and reproductive organs biomass by 47–101% compared with other counterparts. Root protective enzyme and nitrate reductase (NR) activity, potential quantum yield of photosystem (PS) II (*F*_v_*/F*_m_), PSII quantum yield in the light [Y(II)] and electron transport rate of PSII were significantly higher in H_20_W_55_N_1_ prior to 82 days after emergence. Root NR activity and protective enzyme were significantly correlated with chlorophyll, *F*_v_*/F*_m_, Y(II) and stomatal conductance. Hence, shallow irrigation (20 cm) with moderate irrigation and N-fertilization application could increase cotton root NR activity and protective enzyme leading to enhance light capture and photochemical energy conversion of PSII before the full flowering stage. This enhanced photoassimilate to reproductive organs.

## Introduction

Water and nutrient availability has become a major limitation to photosynthate production of crop plants, especially in an arid environment (Lichtenthaler, 1996; Farooq et al., 2009). Moisture stress can decrease nutrient use efficiency and an inappropriate water-nutrient application can degrade soil physicochemical properties (pH, water permeability and nutrient status), resulting in yield loss (Vitousek et al., 1997; Hura et al., 2007). To improve crop performance, it is important to investigate the physiological processes involved in water-nutrient stress adaptation in plants. Therefore, crop management practices such as application of N fertilizer and alternative irrigation systems are often practiced (Farooq et al., 2009; Yahdjian et al., 2011).

Photosynthesis is the most essential process in the plant for growth and biomass production, thus it is the driving force for yield formation (Raines, 2011; Khan et al., 2017). However, low availability of water-nutrient can adversely influence photosynthesis (Lawlor and Cornic, 2002) and photosynthetic pigments (Makoto and Koike, 2007). Water or N stress can also cause a substantial reduction of photosynthetic enzymes (Evans, 1989; Flexas et al., 2006a), lead to disorganization of thylakoid membranes (Ladjal et al., 2000) and inhibition of photosystem activity (Lawlor and Cornic, 2002; Flexas et al., 2006a). Lower CO_2_ assimilation may lead to an imbalance between photochemical activity at PSII and electron requirement for photosynthesis (Hayashi et al., 2013; Yi et al., 2016). These changes can induce reactive oxygen species (ROS) production in leaves (Asada, 1999), which is potentially harmful to PSII reaction centers. This can also decrease the response level of the antioxidant enzymes to ROS (Reddy et al., 2004; Yi et al., 2016). Lower photochemical activity of photosystem is negatively associated with ETR. This can result in an inadequate supply of adenosine triphosphate (ATP) and or nicotinamide adenine dinucleotide phosphate (NADPH) to reductive Calvin-Benson-Bassham (CBB) cycle that limited in ribulose-1,5-bisphosphate carboxylase/oxygenase (RuBP) regeneration (Tezara et al., 1999). Furthermore, this can adversely affect leaf photosynthesis, resulting in lower photosynthate production (Lawlor and Cornic, 2002).

Cotton roots are very sensitive to water or nutrient stress (Einsmann et al., 1999; Wasson et al., 2012), while the root morphological traits can be regulated by water-nutrient modes or rates (Wasson et al., 2012; Wu et al., 2013). Adequate water or N application can improve root distribution in the water or N applied zone in the soil, which increases water and N uptake resulting in higher accumulation of photosynthates (Blum, 2005; Wang et al., 2013; Xu et al., 2015; Zhang et al., 2017). Luo et al. (2014) suggested that cotton root vigor ensures the absorption of water-N and root vigor in the 40–120 cm soil layers and is closely associated with the *P*_n_. In addition, water or N stress negatively affects root physiological characteristics (nitrate reductase, root vigor and hormonal changes) resulting in decreased N uptake (Elvira et al., 2004; Blum, 2005; Luo et al., 2014). It is important to promote vertical distribution of roots for higher leaf photosynthetic capacity (Wu et al., 2013; Luo et al., 2014).

Cotton is the fifth largest fiber-producing plant worldwide. The total seed cotton yield in China accounts for approximately 30% of the worldwide cotton production. Xinjiang is the largest province of China which contributes about 60% of the cotton (China Cotton Fair Examination, 2012/2013). However, Xinjiang is an arid area in northwestern China; cotton production in the area mainly depends on irrigation. To achieve optimal yields, growers have increased water input (the agricultural water input increased by 20% from 1965 to 2000) (Leiwen, 2005) and agricultural nutrient application (the nitrogen rate for the highest yield was up to 300 kg ha^-1^ in Xinjiang, China, whereas the nitrogen rate for the highest cotton lint yield was 240 kg ha^-1^ in the Yangtze River Valley) (Yang et al., 2011a). These practices have resulted in several problems, including nutrient imbalance in the soil (Ndabamenye et al., 2013), declining yields and quality (Loveys et al., 2000; Hu et al., 2011), and increasing soil salinization (Hoorn et al., 1993; Aragüés et al., 2014). It is important to identify the feasibility of optimal irrigation and fertilization to minimize yield reduction and environment pollution. Therefore, we hypothesized that optimizing water-N supply can increase cotton plant biomass accumulation by maintaining leaf photosynthetic capacity and enhancing root growth. The objectives of this were (1) to investigate the impacts of different water-N application depth and rate on cotton root morphological attributes and physiological activity, antioxidant enzyme activity, chlorophyll fluorescence, gas exchange parameters and dry matter production and (2) to determine the quantitative relationship among these factors in terms of improved plant performance. These data will help to develop management strategies for optimal cotton production in northwestern Xinjiang China.

## Materials and Methods

### Description of Experimental Site

The field experiment was conducted in a rain-proof shed (10 m length, 6 m width and 3 m height; the top of shed was covered of transparent polyethylene film, and it was only closed under rainfall conditions) at Shihezi University, Northwestern Xinjiang, China (45°19′N, 74°56′E) during the 2012 growing season. The mean temperatures from April to October were 16.4, 12.5, 23.1, 27.2, 21.2, 18.3, and 10.1°C, respectively. The mean precipitation from April to October was 31.5, 25.5, 30.3, 17.8, 31.8, 15.8, and 10.8 mm, respectively. Mean temperature and relative humidity were recorded by temperature and humidity recorder (TPJ-20, Tuopu Instruments Ltd., Zhejiang, China) during the crop growing season. Seeds of (Xinluzao 33) cultivar were hand-planted in each polyvinyl chloride (PVC) tube (30 cm × 40 cm; diameter × height). The bottom of each tube was covered with a wire mesh to hold the soil. A composite clay loam soil sample was collected from the field station; passed through a 2 mm sieve and then air dried. PVC tubes were filled up to 0.1–1.2 m. Soil physical and chemical analysis produced the following characteristics: soil texture (<0.01 mm, purple clay loam) 54.0%, pH = 7.6, 1.43 g cm^-3^ bulk density, EC 0.53 dS m^-1^, 12.5 g kg^-1^ organic matter, 5.85g kg^-1^ total N, 1.3 g kg^-1^ available N, 1.2 g kg^-1^ total P, 3.54 mg kg^-1^ water P, 16.2 mg kg^-1^ available P, 1.77 g kg^-1^ total K, 195 mg kg^-1^ available K.

### Experimental Design and Field Management

The experiment was laid out in a randomized complete block design with nine treatments as follows: two irrigation levels, i.e., deficit irrigation (irrigation level was maintained at 55 ± 5% of field capacity, W_55_) and adequate irrigation (irrigation level was maintained at 75 ± 5% of field capacity, W_75_); two N fertilizer rate was no nitrogen (N_0_, no nitrogen application) and moderate N (0.216 N g kg^-1^ dry soil, N_1_) and two application depths of 20 cm (H_20_) or 40 cm (H_40_) from soil surface. CK (check or control), irrigation and N fertilizer on the depth of 0 cm from soil surface, irrigation and N fertilizer application rate, respectively, was the same as W_75_ and N_1_ (irrigation and fertilizer management methods widely used in the local area); irrigation and fertilizer management level or depth were used in the following combinations: (1) CK, (2) H_20_W_55_N_0_, (3) H_20_W_55_N_1_, (4) H_20_W_75_N_0_, (5) H_20_W_75_N_1_, (6) H_40_W_55_N_0_, (7) H_40_W_75_N_1_, (8) H_40_W_75_N_0_, (9) H_40_W_75_N_1_.

Irrigation and N application depth were assessed using a drainage tube (diameter 2 cm; length 20 cm in the H_20_ and length 40 cm in the H_40_), while drip irrigation was supplemented to control (CK). This system is widely used in Xinjiang province. N fertilizer was applied at the rate of 0 g tube^-1^ (N_0_) and 2.76 g tube^-1^ (N_1_) treatment. Nitrogen was applied with the ratio of basal fertilizer to topdressing of 1:4. Phosphorus and potassium were supplemented as basal fertilization. Nitrogen in the form of Urea [CO(NH_2_)_2_, 46.0% N] at the rate of 13.8 g per tube was used, while 18 g of monopotassium phosphate [(KH_2_PO_4_) 52.0% P_2_O_5_ and 35.4% K_2_O] was used per tube for the application of aforementioned amounts of P_2_O_5_ and K_2_O. Topdressing N fertilizers were applied in equal amounts at 55, 70, 85, and 100 days after planting.

Four seeds were sown at a depth of 3 cm in each tube on 21 April. The seeds were spaced 10 cm apart in one direction and 20 cm apart in the other. Soil water content was measured after each 3-day interval using Watermarks (Soil Moisture Meter, IRROMETER COMPANY, Inc., NE, United States) at 20, 40, and 60 cm of the soil depth. The Watermarks were installed in each treatment of three replicates in order to indicate the soil water variation. Direct irrigation method was employed for H_20_ and H_40_ treatment to the required depth, while drip irrigation system was supplemented to CK treatment. Drip irrigations systems (Beijing Lvyuan Inc., China) were installed on the top of each tube and one emitter per tube was fixed. The irrigation rates were monitored using both water meter and switch ball valve. The tops of the tube were covered with a polyethylene film to reduce water loss through evaporation. Plants were thinned 15 days after planting to the desired population. In addition, gas exchange, chlorophyll fluorescence, extraction of chlorophyll and protective enzymes in the leaves from the top was performed six times, once every 10 days from 52 DAE to 102 DAE. Protective enzymes and root morphology parameters were measured at 82 and 102 DAE, respectively. Roots less than 5 mm were selected for measuring protective enzymes in the root. Shoot and root samples for MDA contents, antioxidant enzyme activities, free proline content and nitrate reductase were put in a disposable ziplock bag and stored at -80°C. To extract enzymes to test for activity, the samples were put in 8 mL container and then ground using a freezing grinder for 45 s. Cultural management practices, e.g., weeding, hoeing and pesticide application were implemented according to the cotton demand.

### Data Collection and Observations

#### Soil Water Content

Watermark (Soil Moisture Meter, IRROMETER Inc., NE, United States) was used to assess the change of soil water in the 0–60 cm soil layer and maintained at 75% or 55% soil holding capacity in the 0–60 cm soil layer during whole growth period. Water supplied defined as:

(1) $$\begin{array}{c} A\;=\;(W_{p}-W_{a})\;\times \;H \end{array}$$

Where *A* is the volume of water supplied (mm), *W*_p_ is the field capacity in the 0–60 cm soil profile for the experiment. *W*_a_ is the average relative soil moisture content in the 0–60 cm soil profile that was measured using Watermark, and *H* is the thickness of the soil layers using drip irrigation system (mm).

#### Root Growth Measurement

Root distribution was measured in soil columns at 82 and 102 DAE. Each sector (tube) were carefully dug and cut down into 40 cm segments at the top of each column. The segments were immersed in water for 1 h and the roots from each soil layer were rinsed with tap water. Plant debris, weeds, and dead roots were sorted concurrently from ‘live’ roots by hand according to (Gwenzi et al., 2011). Live roots from each sector were evenly spread in a plastic tray containing deionized water and scanned using a flatbed scanner (300 dpi). Root images were analyzed using WinRhizo image analysis software (Regent Instruments, Quebec, Canada). The software was configured to measure RLD and RSD. After scanning, the roots were oven-dried at 80°C for 48 h and root dry mass was weighed to calculate RMD; the RLD, the RSD and the RMD were expressed as cm, cm^2^, and mg per unit volume (cm^3^) of soil, respectively.

#### Proline Content and Nitrate Reductase Activity

Free proline content of cotton leaves was assayed according to the method (Bates et al., 1973). The samples were homogenized in 5 mL of 3% sulfosalicylic acid and centrifuged at 6000 rpm for 10 min. Supernatant was heated with 2 mL of ninhydrin and glacial acetic acid at 100°C for 1 h, respectively. The reaction was further extracted with 4 mL of toluene by vigorously vortexed for 30 s. The absorption of chromophore was determined at 520 nm (Tecan-infinite M200, Switzerland). Nitrate reductase (NR) activity of root and shoot samples were determined by an *in vivo* assay described previously (Radin, 1974).

### Lipid Peroxidation

Lipid peroxidation in cotton roots (including the roots in each soil layer) and leaves were determined as MDA content using the thiobarbituric acid method (Bailly et al., 1996). A 1.0 ml aliquot of supernatant of tissue extract (root or leaf) was mixed with 4 ml of 20% (v/v) trichloroacetic acid containing 0.5% (v/v) thiobarbituric acid. The mixture was heated at 100°C for 30 min, cooled down and centrifuged at 10,000 rpm for 10 min. The absorbance of the supernatant was assayed at 532 and 600 nm.

### Antioxidant Enzymes (SOD, POD, and CAT) Activities in Root and Leaf

The activities of enzymatic antioxidants viz., SOD, POD, and CAT in root and leaf were assessed according to (Zheng et al., 2016) standard procedure. The SOD activity was the amount of extract that gives 50% inhibition in nitrotetrazolium blue chloride (NBT) photoreduction as detected at 560 nm (Tecan-infinite M200, Switzerland). The POD activity was based on the determination of guaiacol oxidation at 470 nm by H_2_O_2_ and was presented as μmol H_2_O_2_ g^-1^ (FW). The change in absorbance at 470 nm was recorded every min by spectrophotometer. One unit of POD activity is the amount of enzyme that causes the decomposition of 1 μg substrate at 470 nm for 1 min in 1 g fresh sample at 37°C. The CAT activity was measured using 0.5 g fresh leaf sample according to Cakmak and Marschner (1992). The CAT activity was defined as the amount of enzyme that causes the decomposition of 1 μmol H_2_O_2_ at 405 nm per min in 1 g fresh sample at 37°C.

### Chlorophyll, Chlorophyll Fluorescence and Leaf Gas Exchange Parameters

All the treatments at each sampled day (for example, 36 samples with four replicates at 52 DAE) were hand grounded and measured using a spectrophotometer within 90 min to avoid the acetone volatilization. Chlorophyll (Chl) *a* and *b* in each sample were extracted from 0.1 g ground fresh leaf mixed with 10 ml of 80% acetone and was measured at 663 and 645 nm according to the method described by Arnon (1949):

(2) $$\begin{array}{c} C_{(Chl\;a}{}_{)}\;=\;12.71D_{663}\;-\;2.59D_{645} \end{array}$$

(3) $$\begin{array}{c} C_{(Chl\;b}{}_{)}\;=\;22.88D_{645}\;-\;4.67D_{663} \end{array}$$

Where *C*_(Chl a)_ or *C*_(Chl b)_ is the content of the Chl *a* or *b*; *D*_663_ or *D*_645_ is the absorbance at 663 or 645 nm using a spectrophotometer (Tecan-infinite M200, Switzerland).

Chlorophyll fluorescence was assessed during diurnal time using a portable saturation pulse fluorometer PAM-2100 equipped with a 2030-B leaf clip holder (Walz, Effeltrich, Germany). Maximal (*F*_m_) and ground (*F*_o_) fluorescence yields of dark-adapted leaves were measured between 05:30 and 06:30. The *F*_o_ was obtained with a measuring light of 0.5 μmol m^-2^ s^-1^ at a frequency of 0.6 kHz, while the *F*_m_ was measured with a 0.8 s saturating pulse at >8,000 μmol m^-2^ s^-1^. Potential quantum yield of PSII (*F*_v_/*F*_m_) was calculated where *F*_v_ is the maximum variable fluorescence (*F*_v_ = *F*_m_ -*F*_o_). During the diurnal time, *F*_s_ (fluorescence of the light-adapted leaf) and *F_m_′* (the maximum light-adapted fluorescence) were measured. The *F*_s_ was obtained at a frequency of 20 kHz; F_m_′ was measured with a 0.8 s saturating pulse at >8,000 μmol m^-2^ s^-1^. The Y(II) was calculated as (F_m_′ -*F*_s_)/F_m_′ (Genty et al., 1989). qP was calculated, i.e., (F_m_′ -*F*_s_)/(F_m_′- F_o_′) according to (Krause and Weis, 1991). Minimal fluorescence under light condition (F_o_′) was assessed by using the equation F_o_′ = *F*_o_/(*F*_v_/*F*_m_ + *F*_o_/F_m_′) as suggested by Oxborough and Baker (1997). qN was determined, i.e., (*F*_m_ - F_m_′)/F_m_′, where *F*_m_ is the value of the predawn measurements using the (Bilger and Björkman, 1990) method. The ETR was assessed using a leaf absorptance of 0.85 and that half of the absorbed light was partitioned to each photosystem: ETR(II) = Y(II) × PPFD × 0.85 × 0.5, in the equation, PPFD incident on the same position of the leaf surface and the leaf was kept under natural conditions, 0.5 indicated that two photons are used for exciting one electron by assuming an equal distribution of excitation between photosystems II and I (Krall and Edwards, 1992), 0.85 is considered the most common leaf absorbance coefficient for C_3_ plants (Björkman and Demmig, 1987) under different environmental conditions and leaf age (Schultz, 1996).

The *P*_n_, *g*_s_ and *C*_i_ were measured between 10:00 and 12:00 h using a photosynthesis system (Li-6400, Li-COR Inc., Lincoln, NE, United States). The light intensity was configured at 1,800 μmol m^-2^ s^-1^ and other parameters varied over a narrow range, [25–32°C, 330–350 μmol (CO_2_) mol^-1^] for all treatments.

### Dry Mass Production

Twelve plants (three PVC tubes) were selected from each treatment and cut down at the cotyledon position after photosynthesis and chlorophyll fluorescence were measured. Plants were carefully dissected into leaves, stems, buds, flowers and bolls. Samples were dried at 80°C for 48 h and weighed for determination of dry mass.

### Statistical Analysis

Analysis of variance (ANOVA) and correlation analysis were performed using SPSS 16.0 software. Differences between treatments were considered significant at *P* < 0.01 according to least significant difference (LSD) tests. The figures were plotted using SigmaPlot 10.0 software.

## Results

### Biomass Accumulation and Allocation

Cotton plant dry matter accumulation was significantly influenced by irrigation modes and N rates. H_20_ compared with H_40_ produced 26.5, 33.7, and 33.4% more total dry mass reproductive organ and leaf biomass accumulation, respectively. In contrast, the root dry matter accumulation was decreased by 15% for H_20_ (**Table 1**) compared to H_40_. Under H_20_ condition, the total dry matter in W_75_N_1_ increased by 29.0, 47.1, 78.1, and 82% than CK, W_75_N_0_, W_55_N_1,_ and W_55_N_0_, respectively, reproductive structures dry matter in W_75_N_1_ were 46.6, 59.0, 91.2, and 101.2% more, while the root biomass in W_75_N_1_ decreased by 2.5, 20.2, 41.1, and 52.7% compared with CK, W_75_N_0_, W_55_N_1_, and W_55_N_0_, respectively.

**Table 1:** The change in leaf biomass (g plant^-1^), stem biomass (g plant^-1^), square and boll biomass (g plant^-1^), total aboveground biomass (g plant^-1^), root biomass (g plant^-1^), total biomass (g plant^-1^), root shoot ratio of cotton plant in coupling of irrigation depth and water-nitrogen application rate.

Treatment	Leaf biomass	Stem biomass	Square and boll biomass	Total aboveground biomass	Root biomass	Total biomass	Root shoot ratio
CK	11.96 ± 0.15^b^	9.42 ± 0.07^b^	17.29 ± 0.39^b^	38.67 ± 0.31^b^	3.0 ± 1.44^c^	41.66 ± 1.3^b^	0.08 ± 0.04^e^
H_20_W_55_N_0_	7.78 ± 0.18^d^	4.66 ± 0.14^f^	12.6 ± 0.12^g^	25.04 ± 0.24^g^	4.46 ± 0.38^b^	29.5 ± 0.51^e^	0.18 ± 0.01^b^
H_20_W_55_N_1_	7.19 ± 0.05^e^	5.61 ± 0.15^e^	13.26 ± 0.13^f^	26.06 ± 0.23^f^	4.12 ± 0.89^bc^	30.18 ± 0.89^e^	0.16 ± 0.03^c^
H_20_W_75_N_0_	9.64 ± 0.91^c^	7.44 ± 0.94^c^	15.94 ± 0.26^c^	33.02 ± 0.3^c^	3.51 ± 0.59^bc^	36.53 ± 0.33^c^	0.11 ± 0.02^d^
H_20_W_75_N_1_	13.4 ± 0.17^a^	12.08 ± 0.07^a^	25.35 ± 0.32^a^	50.83 ± 0.11^a^	2.92 ± 0.6^c^	53.75 ± 0.64^a^	0.06 ± 0.01^f^
H_40_W_55_N_0_	6.11 ± 0.23^f^	4.16 ± 0.15^g^	8.7 ± 0.05^i^	18.97 ± 0.14^i^	5.03 ± 0.71^a^	24 ± 0.85^f^	0.27 ± 0.04^a^
H_40_W_55_N_1_	7.11 ± 1.02^e^	5.35 ± 0.98^e^	11.9 ± 0.11^h^	24.36 ± 0.2^h^	4.61 ± 0.23^b^	28.97 ± 0.38^e^	0.19 ± 0.01^b^
H_40_W_75_N_0_	7.67 ± 0.42^e^	6.32 ± 0.47^d^	14.3 ± 0.17^e^	28.29 ± 0.11^e^	4.11 ± 0.75^bc^	32.4 ± 0.64^d^	0.15 ± 0.03^bc^
H_40_W_75_N_1_	7.56 ± 0.14^e^	6.65 ± 0.24^cd^	15.44 ± 0.03^d^	29.65 ± 0.1^d^	3.51 ± 0.44^bc^	33.15 ± 0.45^d^	0.12 ± 0.01^cd^
H	^∗∗^	^∗∗^	^∗∗^	^∗∗^	^∗^	^∗∗^	^∗∗^
W	^∗∗^	^∗∗^	^∗∗^	^∗∗^	^∗^	^∗∗^	^∗∗^
N	^∗∗^	^∗∗^	^∗∗^	^∗∗^	ns	^∗∗^	^∗∗^
H × W	^∗∗^	^∗∗^	^∗∗^	^∗∗^	ns	ns	^∗∗^
H × N	^∗∗^	^∗∗^	^∗∗^	^∗∗^	ns	ns	^∗∗^
W × N	^∗∗^	^∗∗^	^∗∗^	^∗∗^	ns	ns	^∗∗^
H × W × N	^∗∗^	^∗∗^	^∗∗^	^∗∗^	ns	^∗^	^∗∗^

*Data are mean ± SE, different letters indicate a significant difference (p < 0.05) among the treatments. ^∗∗, ∗^, and ns indicate extremely significant differences (p < 0.01), significant (p < 0.05) and no significant difference (p > 0.05), among watered depth, watered rate and nitrogen application rate within an irrigation treatment. H_*20*_, watered depth at 20 cm soil layer; H_*40*_, watered depth at 40 cm soil layer; W_*75*_, water application rates with 75% field capacity; W_*55*_, water application rates with 55% field capacity; N_*1*_, nitrogen application; N_*0*_, no nitrogen application; CK, surface irrigation, the irrigated rate of 75% field capacity and nitrogen application*.

### The Enzymatic Activity and Pigment Content in Leaf

**FIGURE 1:**
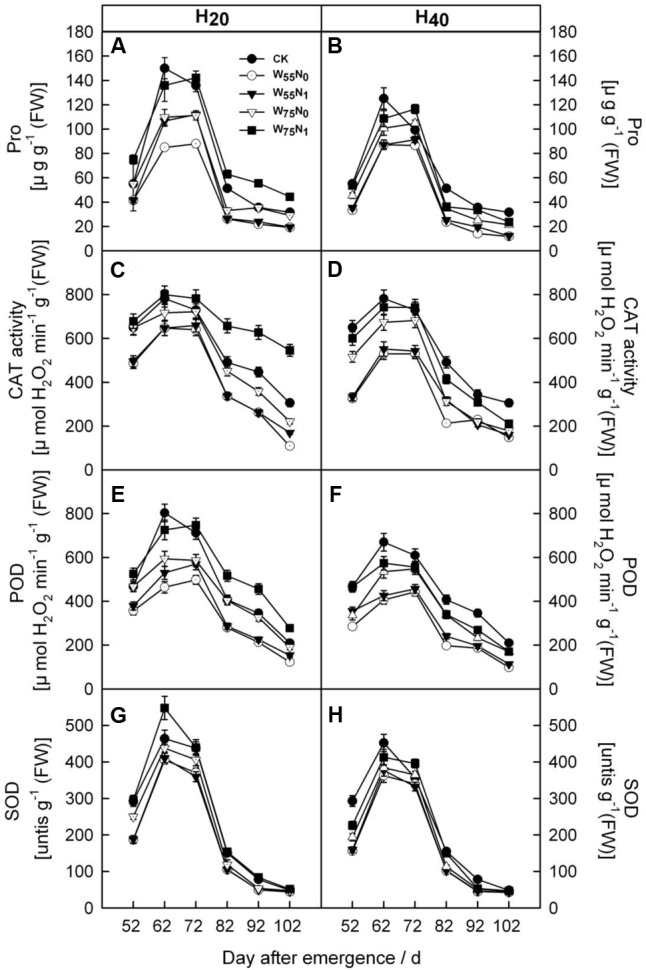
Changes in **(A,B)** proline [Pro, μg g^-1^ (FW)], **(C,D)** catalase [CAT, μmol H_2_O_2_ min^-1^ g^-1^ (FW)], **(E,F)** peroxidase [POD, μmol H_2_O_2_ min^-1^ g^-1^ (FW)] and **(G,H)** superoxide dismutase (SOD, [units g^-1^ (FW)] in leaf of cotton plant in coupling of irrigation depth and water-nitrogen application rate during 52 to 102 days after emergence. Data are mean ± SE. H_20_, watered depth at 20 cm soil layer; H_40_, watered depth at 40 cm soil layer; W_75_, water application rates with 75% field holding capacity; W_55_, water application rates with 55% field capacity; N_1_, nitrogen application; N_0_, no nitrogen application; CK, surface irrigation, the irrigated rate of 75% field capacity and nitrogen application.

**FIGURE 2:**
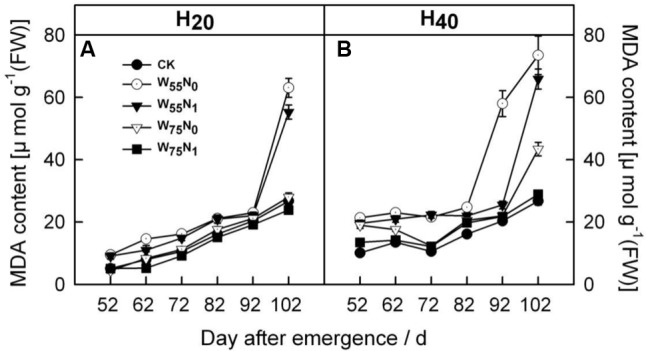
Changes in **(A,B)** malondialdehyde (MDA, μmol g^-1^ FW) in leaf of cotton plant in coupling of irrigation depth and water-nitrogen application rate during 52 to 102 days after emergence. Data are mean ± SE. H_20_, watered depth at 20 cm soil layer; H_40_, watered depth at 40 cm soil layer; W_75_, water application rates with 75% field capacity; W_55_, water application rates with 55% field capacity; N_1_, nitrogen application; N_0_, no nitrogen application; CK, surface irrigation, the irrigated rate of 75% field capacity and nitrogen application.

With the plant development cotton leaf Pro (**Figures 1A,B**), CAT (**Figures 1C,D**), POD (**Figures 1E,F**), and SOD (**Figures 1G,H**) firstly rose and then declined later in the season. Cotton leaf Pro, CAT, POD, and SOD were increased by 6.2–19.9, 20.7–26.8, 17.8–240.4, 40–344.4, 35.0–110.1, 58.9–186.8, 2.0–5.5, and 4.0–44.0 in H_20_ compared with CK and H_40_. The Pro, CAT, POD, and SOD declined in the following order, W_75_N_1_, W_55_N_1_, W_75_N_0_, and W_55_N_0_ under both H_20_ and H_40_. Leaf MDA content was increased as the plant grew, the MDA accumulation in H_20_ was 1.1–2.7 and 2.6–8.4 lower, respectively, compared with CK and H_20_ during whole growth season (**Figure 2**).

**FIGURE 3:**
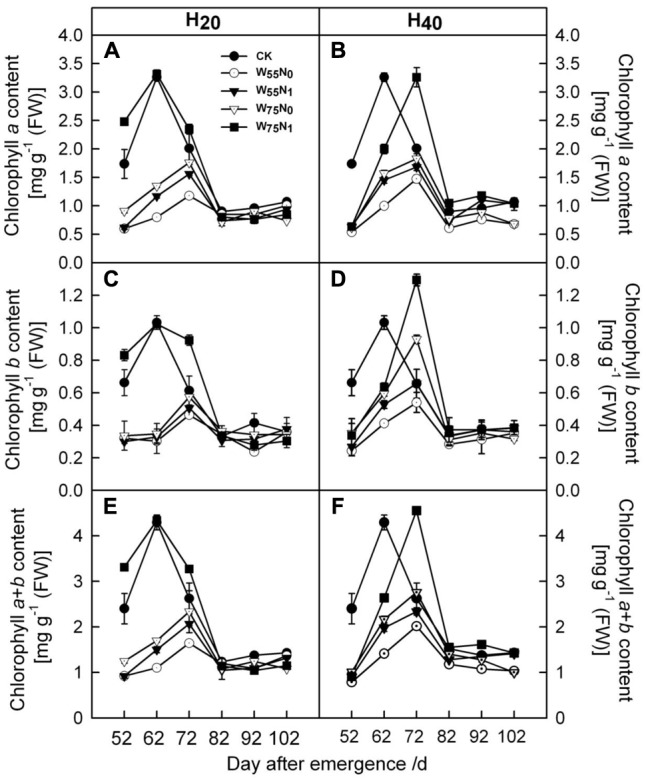
Changes in **(A,B)** chlorophyll *a* [mg g^-1^ (FW)], **(C,D)** chlorophyll *b* [mg g^-1^ (FW)] and **(E,F)** chlorophyll *a*+*b* [mg g^-1^ (FW)] in leaf of cotton plant in coupling of irrigation depth and water-nitrogen application rate during 52 to 102 days after emergence. Data are mean ± SE. H_20_, watered depth at 20 cm soil layer; H_40_, watered depth at 40 cm soil layer; W_75_, water application rates with 75% field capacity; W_55_, water application rates with 55% field capacity; N_1_, nitrogen application; N_0_, no nitrogen application; CK, surface irrigation, the irrigated rate of 75% field capacity and nitrogen application.

As the plant grew, cotton leaf Chl *a* and *b* increased but decreased later in the season (**Figure 3**). The Chl *a* and *b* content was 0.05–0.74 and 1.31–1.84 more in H_20_ than H_40_ before 82 DAE. H_20_ combined with W_75_N_1_ resulted in higher Chl *a* and *b* content compared with other combinations.

### Chlorophyll Fluorescence, Photosystem II and Leaf Gas Exchange Parameters

Most of the chlorophyll fluorescence, PSII and gas exchange parameters of cotton leaves were significantly influenced by irrigation methods and N levels.

**FIGURE 4:**
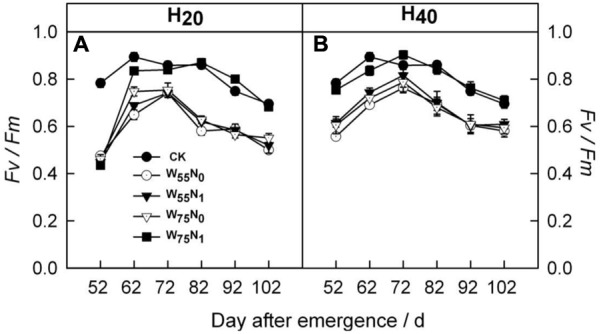
Changes in **(A,B)** potential quantum yield of photosystem II (*F*_v_*/F*_m_) of cotton plant in coupling of irrigation depth and water-nitrogen application rate during 52 to 102 days after emergence. Data are mean ± SE. H_20_, watered depth at 20 cm soil layer; H_40_, watered depth at 40 cm soil layer; W_75_, water application rates with 75% field capacity; W_55_, water application rates with 55% field capacity; N_1_, nitrogen application; N_0_, no nitrogen application; CK, surface irrigation, the irrigated rate of 75% field capacity and nitrogen application.

The H_20_ treatment exhibited 6.8 and 3.2% increment in *F*_v_/*F*_m_ compared with CK and H_40_ after 72 DAE (**Figure 4**). The H_20_W_75_N_1_ combination had higher *F*_v_*/F*_m_ followed by other counterparts in H_20_ and H_40_ conditions after 72 DAE.

**FIGURE 5:**
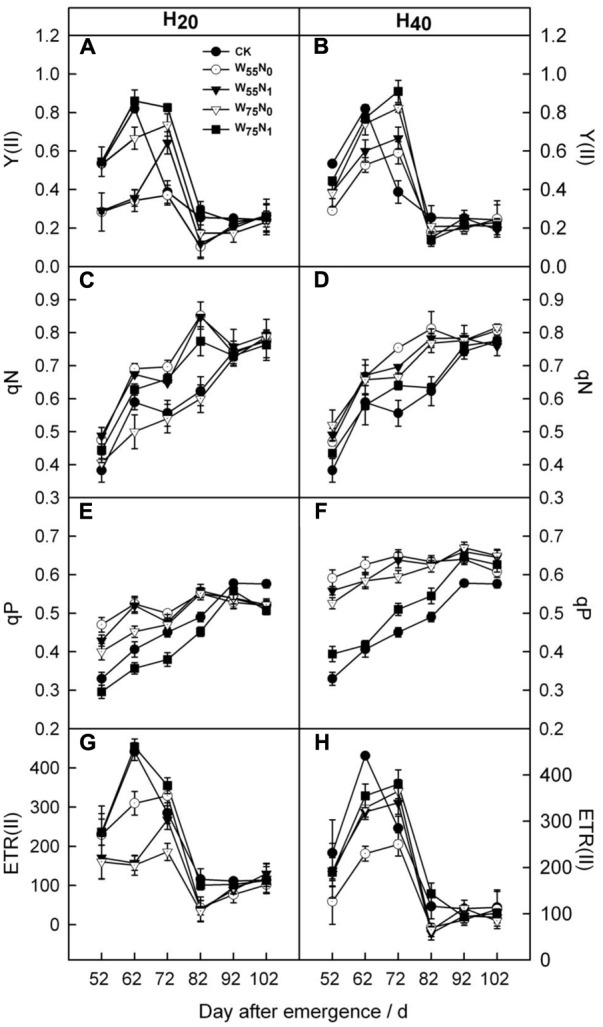
Changes in **(A,B)** PSII quantum yield in the light [Y(II)], **(C,D)** non-photochemical quenching coefficient (qN), **(E,F)** photochemical quenching coefficient (qP) and **(G,H)** the electron transport rate of PSII [ETR(II)] of cotton plant in coupling of irrigation depth and water-nitrogen application rate during 52 to 102 days after emergence. Data are mean ± SE. H_20_, watered depth at 20 cm soil layer; H_40_, watered depth at 40 cm soil layer; W_75_, water application rates with 75% field capacity; W_55_, water application rates with 55% field capacity; N_1_, nitrogen application; N_0_, no nitrogen application; CK, surface irrigation, the irrigated rate of 75% field capacity and application nitrogen.

The Y(II) and ETR(II) were increased by 0.011–0.44 and 0.09–0.15, and 4.6–70 and 46.3–99.0 in H_20_ compared with CK and H_40_ before 82 DAE (**Figure 5**). In contrast, qN and qP was decreased under H_20_ conditions during whole growth season. In addition, H_20_W_75_N_1_ resulted in highest Y(II) and ETR(II) compared with other combinations before 82 DAE.

**FIGURE 6:**
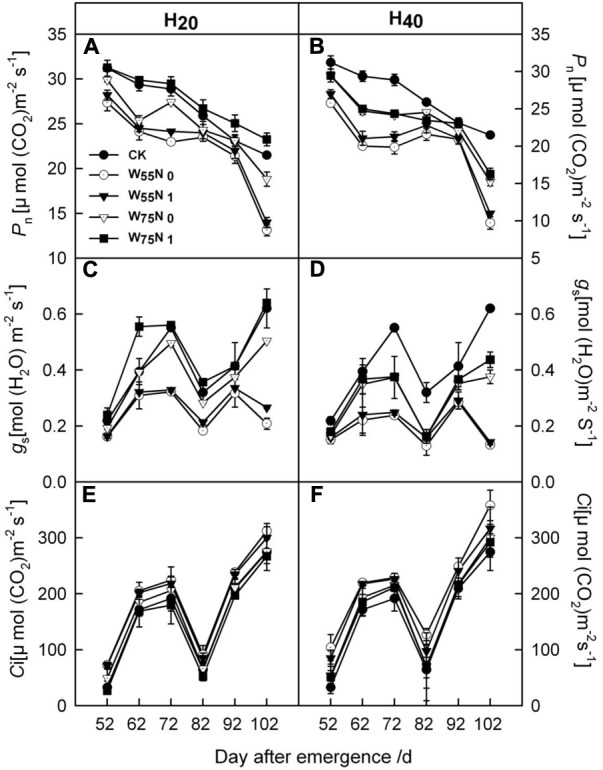
Changes in **(A,B)** the net photosynthetic rate [*P*_n_, μmol (CO_2_) m^-2^ s^-1^], **(C,D)** stomatal conductance [*g*_s_, mol (H_2_O) m^-2^ s^-1^] and **(E,F)** intercellular CO_2_ concentration [*C*_i_, μmol (CO_2_) m^-2^ s^-1^] in leaf of cotton plant in coupling of irrigation depth and water-nitrogen application rate during 52 to 102 days after emergence. Data are mean ± SE. H_20_, watered depth at 20 cm soil layer; H_40_, watered depth at 40 cm soil layer; W_75_, water application rates with 75% field capacity; W_55_, water application rates with 55% field capacity; N_1_, nitrogen application; N_0_, no nitrogen application; CK, surface irrigation, the irrigated rate of 75% field capacity and application nitrogen.

Most of the cotton leaf gas exchange parameters, i.e., *P*_n_, *g*_s_ were increased by 0.06–1.93 and 1.8–7.0, and 0.01–1.6 and 0.06–0.2 in the H_20_ compared with CK and H_40_ during whole growth season (**Figure 6**). In contrast, *C*_i_ was higher in H_40_ treatment than in H_20_ and CK during whole growth season. H_20_W_75_N_1_ exhibited highest values for *P*_n_ and *g*_s_ compared with other treatments during the whole growth season.

### Root Morphological Features

**FIGURE 7:**
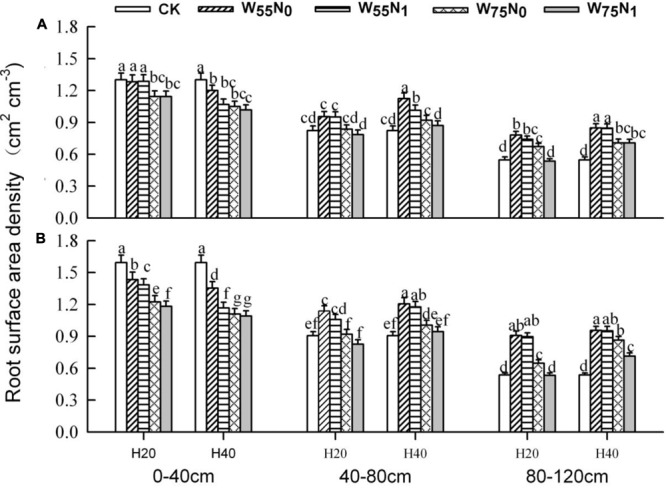
Changes in root surface area density (RSD, cm^2^ cm^-3^) of cotton plant in coupling of irrigation depth and water-nitrogen application rate at 82 days **(A)** and 102 days **(B)** after emergence. Data are mean ± SE, different letters indicate a significant difference (*p* < 0.05) among the treatments in one soil layer at 82 days or 102 days. H_20_, watered depth at 20 cm soil layer; H_40_, watered depth at 40 cm soil layer; W_75_, water application rates with 75% field holding capacity; W_55_, water application rates with 55% field capacity; N_1_, nitrogen application; N_0_, no nitrogen application; CK, surface irrigation, the irrigated rate of 75% field capacity and nitrogen application.

**FIGURE 8:**
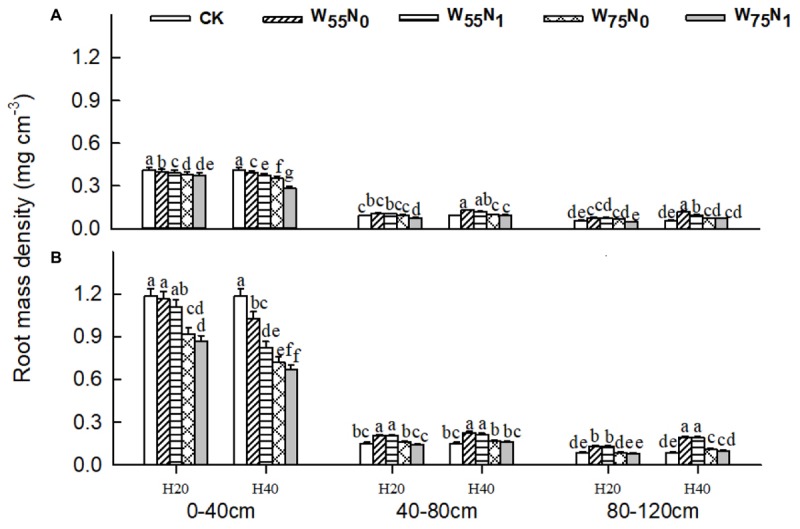
Changes in root mass density (RMD, mg cm^-3^) of cotton plant in coupling of irrigation depth and water-nitrogen application rate at 82 days **(A)** and 102 days **(B)** after emergence. Data are mean ± SE, different letters indicate a significant difference (*p* < 0.05) among the treatments in one soil layer at 82 days or 102 days. H_20_, watered depth at 20 cm soil layer; H_40_, watered depth at 40 cm soil layer; W_75_, water application rates with 75% field capacity; W_55_, water application rates with 55% field holding capacity; N_1_, nitrogen application; N_0_, no nitrogen application; CK, surface irrigation, the irrigated rate of 75% field capacity and nitrogen application.

**FIGURE 9:**
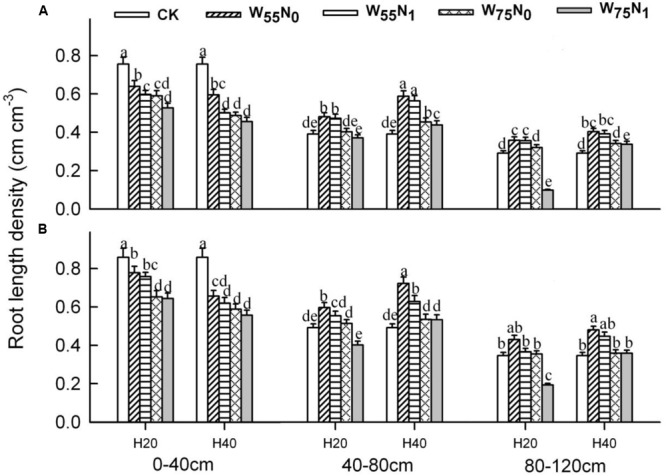
Changes in root length density (RLD, cm cm^-3^) of cotton plant in coupling of irrigation depth and water-nitrogen application rate at 82 days **(A)** and 102 days **(B)** after emergence. Data are mean ± SE, different letters indicate a significant difference (*p* < 0.05) among the treatments in one soil layer at 82 days or 102 days. H_20_, watered depth at 20 cm soil layer; H_40_, watered depth at 40 cm soil layer; W_75_, water application rates with 75% field capacity; W_55_, water application rates with 55% field holding; N_1_, nitrogen application; N_0_, no nitrogen application; CK, surface irrigation, the irrigated rate of 75% field holding capacity and nitrogen application.

The plants grown in the 0–40 cm soil layer with H_20_ was 11.0–13.0% and 15.6–16.9% and 11.8–25.1% higher for RSD, RLD, and RMD, respectively, at 82 and 102 DAE, compared with H_40_ (**Figures 7–9**); in the 40–120 cm soil layer, RSD, RLD, and RMD were 12.8–12.5%, 19.2–23.0% and 1.9–19.6% lower for H_20_ compared with H_40_. Under H_20_ or H_40_ conditions, RSD and RLD decreased in the order W_55_N_0_, W_75_N_0_, W_55_N_1_, and W_75_N_1_.

### Root Physiological Features

In the 0–80 cm soil layer, the CAT, POD, and SOD activity were significantly higher in H_20_ compared with H_40_ (**Table 2**), although the MDA showed a reverse trend. Cotton roots proline (Pro) content, CAT, POD, and SOD activity declined under H_20_ or H_40_ condition in the following order, W_75_N_1_, CK, W_55_N_1_, W_75_N_0_, and W_55_N_0_, while MDA rose in the 0–80 cm soil layer at 82 and 102 DAE.

**Table 2:** Changes in superoxide dismutase [SOD, units g^-1^ (FW)], catalase [CAT, μmol H_2_O_2_ min^-1^g^-1^ (FW)], peroxidase [POD, μmol H_2_O_2_ min^-1^g^-1^ (FW)] and malondialdehyde [MDA, μmol g^-1^ (FW)] in root of cotton plant for irrigation depth and water-nitrogen application rate at 82 and 102 days after emergence.

Soil layer	Treatments		82 days after emergence				102 days after emergence			
			SOD	CAT	POD	MDA	SOD	CAT	POD	MDA
0–40		CK	40.2 ± 2.1^a^	200.8 ± 10.3^b^	169.9 ± 7.9^b^	22.4 ± 1^e^	5.1 ± 0.2^ab^	117.5 ± 5.2^b^	54.1 ± 2.2^b^	27.3 ± 1.1^e^
	H_20_	W_55_N_0_	29.2 ± 1.5^cde^	96.1 ± 4.8^f^	76 ± 3.4^f^	46 ± 2.2^bc^	1.8 ± 0.1^f^	54.6 ± 2.3^e^	26.4 ± 1.3^f^	67.3 ± 3.3^b^
		W_55_N_1_	32.3 ± 1.3^bcd^	97.6 ± 4.9^ef^	89.8 ± 4.6^e^	42.7 ± 2.1^cd^	2.4 ± 0.1^e^	66.8 ± 3.4^d^	37 ± 1.8^e^	52.3 ± 2.6^c^
		W_75_N_0_	34.8 ± 1.7^b^	117.2 ± 4.3^c^	168.9 ± 7.5^b^	38 ± 1.5^d^	5 ± 0.2^bc^	92.7 ± 4.5^c^	47.3 ± 2.4^c^	29.8 ± 1.3^e^
		W_75_N_1_	40.5 ± 2.1^a^	264.1 ± 12.6^a^	222.7 ± 4.9^a^	11.1 ± 0.6^f^	5.5 ± 0.2^a^	131.7 ± 6.2^a^	62.5 ± 3^a^	14.7 ± 0.6^f^
	H_40_	W_55_N_0_	24.7 ± 1.3^e^	48.7 ± 2.5^h^	57 ± 2.6^g^	69.8 ± 2.5^a^	1.1 ± 0.1^g^	34.6 ± 1.2^f^	13.7 ± 0.6^g^	118.8 ± 5.5^a^
		W_55_N_1_	28.8 ± 1.3^de^	84.1 ± 4.1^g^	72.8 ± 3.3^f^	48.7 ± 2.3^b^	1.1 ± 0.1^g^	51.8 ± 2.6^e^	16.3 ± 1.5^g^	67.4 ± 3.4^b^
		W_75_N_0_	33.6 ± 1.7^bc^	106.2 ± 4.3^de^	101.4 ± 5.5^d^	42 ± 1.1^cd^	4.2 ± 0.2^d^	70.7 ± 3.2^d^	39.3 ± 1.5^de^	47.6 ± 2.3^cd^
		W_75_N_1_	34 ± 1.7^b^	112 ± 5.3^cd^	149.5 ± 6.5^c^	41.7 ± 2.1^cd^	4.5 ± 0.2^cd^	88.8 ± 3.8^c^	42.8 ± 2.3^cd^	42.8 ± 2.1^d^
40–80		CK	44.6 ± 2.1^a^	249.4 ± 9.5^b^	248 ± 13.3^b^	9.1 ± 0.4^h^	6.8 ± 0.3^ab^	217.5 ± 10.8^b^	228.7 ± 7.5^b^	24.1 ± 1.5^d^
	H_20_	W_55_N_0_	32.7 ± 1.6^cd^	97.3 ± 4.4^d^	156.1 ± 7.9^f^	33.5 ± 1.6^c^	2.1 ± 0.1^f^	74.7 ± 3^de^	61.7 ± 0.3^f^	54.5 ± 2.4^b^
		W_55_N_1_	32.7 ± 1.6^cd^	105.8 ± 5.7^cd^	199.3 ± 6.7^e^	24.4 ± 1.2^d^	3.2 ± 0.1^e^	76 ± 3.1^de^	89.4 ± 4.4^e^	51.6 ± 2.6^b^
		W_75_N_0_	38 ± 1.5^b^	126.2 ± 5.2^c^	216.3 ± 10.2^c^	15.1 ± 0.6^g^	5.9 ± 0.3^bc^	101.3 ± 4.5^c^	166.6 ± 8.2^c^	24.9 ± 1.2^d^
		W_75_N_1_	45.4 ± 2.2^a^	347.7 ± 14.8^a^	280.3 ± 13.3^a^	5.7 ± 0.2^i^	6.9 ± 0.3^a^	269.9 ± 12.9^a^	254.4 ± 11.9^a^	10.7 ± 0.5^e^
	H_40_	W_55_N_0_	31.5 ± 1.5^d^	64.5 ± 3.1^e^	65.2 ± 2.8^g^	39.8 ± 1.9^a^	1.6 ± 0.7^f^	62 ± 3.6^e^	22.3 ± 1.1^g^	86.1 ± 4.3^a^
		W_55_N_1_	31.7 ± 1.6^d^	95.7 ± 4.4^d^	153.9 ± 5.4^f^	36.7 ± 1.8^b^	1.6 ± 0.5^f^	73.8 ± 3.7^de^	36.7 ± 1.6^g^	55.6 ± 2.3^b^
		W_75_N_0_	37.3 ± 1.7^bc^	111.3 ± 5.2^cd^	203.8 ± 10.1^de^	20.1 ± 1.1^e^	4.4 ± 0.2^d^	80.8 ± 3.4^d^	114.3 ± 5.7^d^	41.2 ± 2.1^c^
		W_75_N_1_	37.8 ± 1.7^b^	115.4 ± 5^cd^	212.5 ± 9.5^cd^	17.1 ± 0.9^f^	5.4 ± 0.2^c^	89.2 ± 4.5^cd^	166.1 ± 8.6^c^	39.9 ± 1.9^c^
80–120		CK	46.7 ± 2.1^ab^	379.6 ± 18.7^a^	277.9 ± 13.1^ab^	6.2 ± 0.3^f^	8 ± 0.4^a^	276.2 ± 13.6^a^	234.7 ± 11.1^b^	11.9 ± 0.6^de^
	H_20_	W_55_N_0_	41.8 ± 2.1^bc^	180.5 ± 9.1^de^	227.8 ± 11.4^cd^	15.7 ± 0.7^c^	2.7 ± 0.1^e^	117.5 ± 5.7^d^	78.1 ± 3.6^e^	20.9 ± 0.8^c^
		W_55_N_1_	43.2 ± 2.2^abc^	198.1 ± 9.5^cd^	248.1 ± 11.7^bcd^	13.9 ± 0.6^cd^	3.8 ± 0.1^d^	118.3 ± 4.9^d^	117.5 ± 5.6^d^	20.2 ± 1^c^
		W_75_N_0_	44.4 ± 2.2^abc^	251.9 ± 12.7^b^	267.8 ± 14^ab^	9.3 ± 0.5^e^	6.1 ± 0.3^b^	217 ± 6.7^b^	226.3 ± 11.5^b^	12.9 ± 0.6^de^
		W_75_N_1_	49 ± 2.4a	381.7 ± 19.2^a^	288.3 ± 12.5^a^	2.1 ± 0.1^g^	8.4 ± 0.4^a^	285 ± 14.5^a^	272.4 ± 13.5^a^	8.5 ± 0.4^e^
	H_40_	W_55_N_0_	34.5 ± 1.7^d^	88.6 ± 4^f^	122.7 ± 5.6^e^	22.7 ± 1.4^a^	2 ± 0.1^f^	79.8 ± 3.6^e^	48.7 ± 2.2^f^	84.4 ± 4.4^a^
		W_55_N_1_	40 ± 1.6^cd^	161.2 ± 7.8^e^	220.6 ± 11.6^d^	18.2 ± 0.9^b^	2 ± 0.1^f^	109.3 ± 5.2^d^	63.2 ± 3.0^ef^	39.5 ± 2^b^
		W_75_N_0_	43.4 ± 2.2^abc^	206.9 ± 10.2^cd^	252.2 ± 12.2^bcd^	12.2 ± 0.7^d^	4.8 ± 0.2^c^	169.5 ± 7.5^c^	122.3 ± 6.2^d^	14.8 ± 0.7^d^
		W_75_N_1_	43.8 ± 2.2^abc^	228.2 ± 10.9^bc^	259.8 ± 13.5^abc^	9.9 ± 0.5^e^	5.6 ± 0.3^b^	209.9 ± 10.5^b^	184.5 ± 8.9^c^	14.5 ± 0.7^d^

*Data are mean ± SE, different letters indicate a significant difference (p < 0.05) between the treatments in one soil layer at 82 days or 102 days. H_*20*_, watered depth at 20 cm soil layer; H_*40*_, watered depth at 40 cm soil layer; W_*75*_, water application rates with 75% field capacity; W_*55*_, water application rates with 55% field capacity; N_*1*_, nitrogen application; N_*0*_, no nitrogen application; CK, surface irrigation, the irrigated rate of 75% field capacity and nitrogen application*.

**FIGURE 10:**
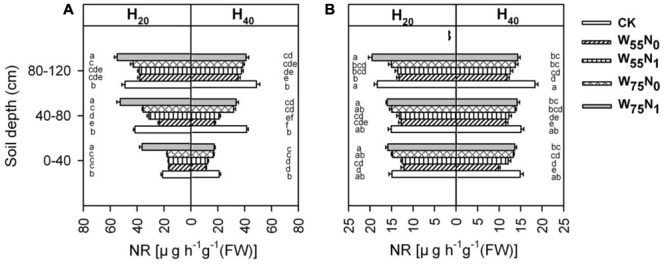
Changes in nitrate reductase [NR, ug h^-1^g^-1^ (FW)] in roots of cotton plant in coupling of irrigation depth and water-nitrogen application rate at 82 days **(A)** and 102 days **(B)** after emergence. Data are mean ± SE, different letters indicate a significant difference (*p* < 0.05) among the treatments in one soil layer at 82 days or 102 days. H_20_, watered depth at 20 cm soil layer; H_40_, watered depth at 40 cm soil layer; W_75_, water application rates with 75% field capacity; W_55_, water application rates with 55% field capacity; N_1_, nitrogen application; N_0_, no nitrogen application; CK, surface irrigation, the irrigated rate of 75% field capacity and nitrogen application.

Root NR was significantly influenced by the study factors (**Figure 10**). NR activity of root was significantly higher for H_20_ compared with H_40_ in whole soil layer at 82 and 102 DAE. Under H_20_ conditions, the NR activity was 21.6, 37.9, 53.8, and 66.5% higher for W_75_N_1_ compared with CK, W_75_N_0_, W_55_N_1_, and W_55_N_0_ in whole soil layer at 82 and 102 DAE, respectively. Under H_40_ condition, the NR activity increased by 17.8, 23.1, 48.0, and 67.2% in CK compared with W_75_N_1_, W_75_N_0_, W_55_N_1_, and W_55_N_0_ in whole soil layer at 82 and 102 DAE, respectively.

### The Relationship of Root with *F*_v_*/F*_m_, Y(II) and *g*_s_

The correlation analysis (**Table 3**) showed that the cotton root NR activity was negatively related (*P* < 0.01) to MDA and was positively related to *F*_v_*/F*_m_ (*P* < 0.01), PSII (*P* < 0.01), total chlorophyll (*P* < 0.05) and *g*_s_ (*P* < 0.01). The MDA was negatively associated (*P* < 0.01) with *F*_v_*/F*_m_, PSII and total chlorophyll content. The RLD significantly positively (*P* < 0.05) related to *g*_s_. Moreover, the *F*_v_*/F*_m_ positively related to PSII (*P* < 0.01), total chlorophyll (*P* < 0.01) and *g*_s_ (*P* < 0.05). These finding implied that improving the root activity and root length contributed to enhanced leaf photosynthesis.

**Table 3:** Relationship among nitrate reductase [NR, μg h^-1^g^-1^ (FW)] and malondialdehyde [MDA, μmol g^-1^ (FW)] in root, root surface area density (RSD, cm^2^cm^-3^), root mass density (RMD, mg cm^-3^), root length density (RLD, cm cm^-3^) and potential quantum yield of photosystem II (*F*_v_*/F*_m_), PSII quantum yield in the light [Y(II)], the electron transport rate of PSII, chlorophyll *a* [mg g^-1^ (FW)] and stomatal conductance [*g*_s_, mol (H_2_O) m^-2^ s^-1^] in leaf of cotton plant at 82 and 102 days after emergence in coupling of irrigation depth and water-nitrogen application rate.

	NR	MDA	RSD	RMD	RLD	*Fv/Fm*	PSII	Chlorophyll	*g*_s_
NR	1.000	–0.871**	0.061	0.057	0.072	0.600**	0.647**	0.479*	0.820**
MDA	–	1.000	–0.027	–0.006	–0.188	–0.552**	–0.496**	–0.177	–0.856**
RSD	–	–	1.000	0.849**	0.938**	–0.152	0.187	0.036	0.342
RMD	–	–	–	1.000	0.794**	–0.254	0.327	–0.283	0.357
RLD	–	–	–	–	1.000	–0.055	0.169	0.043	0.413*
*F*_v_*/F*_m_	–	–	–	–	–	1.000	0.651**	0.568**	0.472*
PSII	–	–	–	–	–	–	1.000	0.189	0.634**
Chlorophyll	–	–	–	–	–	–	–	1.000	0.266
*g*_s_	–	–	–	–	–	–	–	–	1.000

*^∗∗^ and ^∗^, respectively, indicated highly significant differences (*p* < 0.01) and significant differences (*p* < 0.05).*

## Discussion

In modern agricultural systems, water-nutrient management are cotton yield increment technologies especially in arid climatic conditions. The main objective of this study was to determine the effects of irrigation and nitrogen management under different soil depth on cotton crop physiological attributes. In the present study, H_20_W_75_N_1_ increased photosynthate accumulation of aerial part of cotton plant that promoted the allocation of assimilates toward reproductive organs. While the assimilate formation is closely related with root system [root distribution and physiological activity (root vigor and root NR)] and photosystem (light capturing organ, the activity of PSII reactive center and the utilization or consumption of photochemical energy). However, the root system and leaf photosystem act together to promote water-nutrient use efficiency and achieve optimal yield.

Higher root distribution vigor and NR are dependent on water and N absorption (Luo et al., 2015; Gao and Lynch, 2016). In addition, water-N application can also affect root growth or distribution (Jackson et al., 1990; Wasson et al., 2012). We found that H_20_W_75_N_1_ increased root diameter resulted in the development of effective roots (diameter less than 0.05 mm) to efficiently uptake and transport. This protected the integrity of the lipid membrane in the root and enhanced root stress tolerance in the 0–80 cm soil layers at 82 and 102 DAE. A possible reason was that H_20_W_75_N_1_ increased the available water-N in the soil layer, while higher available water-N also promoted the NR content and the root protective enzyme which resulted in more efficient water and N uptake (Jackson et al., 1990). H_20_W_75_N_1_ combination increased antioxidant enzymes content which resulted in lower ROS production in the cotton roots, and higher water-N uptake. Our data is consistent with Medici et al. (2004) and Reddy et al. (2004), which suggested that higher available water-N avoided the water-N stress that causes ROS production in the root.

Application of water-N initially contacts with the root system which in turn affects photosynthesis through root system activity. We observed that total chlorophyll had a positive relationship with the NR in the roots. In contrast, NR activity had a significantly negative relationship with MDA content. This indicated that the chlorophyll in leaf was closely associated to N uptake. This might be due to H_20_W_75_N_1_ resulting in increased available water and N in the soil and decreased the transportation resistance to water-nutrient in roots (Segal et al., 2008; Wasson et al., 2012). Concurrently, inducing NR generation further promoted N absorption and transportation to the aerial parts of the cotton plant (Lilley and Kirkegaard, 2011; Hunter and Ruffner, 2015) prior to 82 DAE. More N accumulation in leaf enhanced chlorophyll formation and conversion into chlorophyll under light conditions (Benli et al., 1991; Armstrong et al., 1995). Furthermore, antioxidants enzymes activity was increased in the leaf prior to 82 DAE and decreased the accumulation of ROS which in turn retarded in the decomposition of the Chl *a* and *b* (Nagata et al., 2005). Our data is inconsistent with the previous research that available water-N in the soil and N content in the leaf did not affect the sorghum crop leaf chlorophyll content (Zhao et al., 2005). The soil properties could have caused lower available water-N in the soil profile. Thus, H_20_W_75_N_1_ combination enhanced water and N as well as the activity of enzymes, which promoted the formation of chlorophyll in leaves prior to 82 DAE.

Measurements of Y(II) provide a rapid method to determine the PSII operating efficiency under different light and environmental conditions (Baker, 2008). In the present study, greater value of Y(II) in different treatments were observed at 62 or 72 DAE, which are considered the most vigorous growth stages of cotton (Yi et al., 2014). The ratios of Y(II) and *P*_n_ was also increased significantly because the Y(II) was increased more quickly than *P*_n_. This increment was accompanied by an increase in the levels of antioxidants and activities of enzymes involved in scavenging ROS led to higher electron flux to oxygen and promoted CO_2_ assimilation. Several researchers also showed similar phenomenon (Fryer et al., 1998; Farage et al., 2006; Baker, 2008). Leaf Pro, CAT, POD, and SOD were increased in H_20_W_75_N_1_ prior to 82 DAE. This increment might be due to the enhanced leaf physiological activity which promoted the antioxidant activity and decreased ROS production and lipid membrane peroxidation. This resulted in higher chlorophyll content and avoided photo inhibition leading to higher PSII activity (Nishiyama et al., 2006; Gill and Tuteja, 2010). Moreover, *F*_v_/*F*_m_ was linearly associated with total chlorophyll which indicates the activity of PSII had a positive relationship with available water-nutrient. Results imply that H_20_W_75_N_1_ enhanced activity of the reaction centers of PSII and the photo protective mechanism of PSII, which promoted photochemical energy conversion and the ETR of PSII prior to 82 DAE. A possible reason might be due to water soluble-nutrient application which also promoted the root absorptive capacity and led to higher leaf N accumulation. This increment in N accumulation contributed to higher Chl *a* and *b* contents prior to 82 DAE (Jeon et al., 2006). On the other hand, relative higher root growth activity ensured higher water soluble-N uptake (Segal et al., 2008; Wasson et al., 2012). This decreased the photo inhibition of PSII and promoted the photochemical efficiency and the ETR of PSII (Jia et al., 2008; Hayashi et al., 2013; Luo et al., 2016).

The higher activity of the reaction center of photosystems drives chemical energy storage and promotes the regeneration of RuBP which led to higher *P*_n_ (Hendrickson et al., 2004; Takahashi et al., 2013). The *P*_n_ showed strong responses to water and N supply (Flexas et al., 2006b; Makoto and Koike, 2007). In this study the *P*_n_ was at peak under H_20_W_75_N_1_ application which was attributed to higher protective enzyme content and NR activity in the roots occasioned by efficient water soluble-nutrient uptake. In addition, more available water-N enhanced absorption and accumulation of NO_3_^-^, NH_4_^+^, K^+^ in the root or leaf has led to efficient water uptake (Lopes and Araus, 2006). Water-N supply decreases water soluble-nutrient stress causing root-sourced signal (ABA), leading to stomatal opening and increased leaf water potential (Siddique et al., 1990; Tardieu and Davis, 1993) and physiological activity in leaves (Blum and Johnson, 1993). We also observed that, H_20_W_75_N_1_ still maintained a greater *P*_n_, and the leaves had begun to decrease the regulatory ability of the stomata. A possible reason was that the leaf senescence caused a lower regulatory ability in the stomata and had a direct thermodynamic effect when the leaf was exposed to higher temperature (about 38 ± 2°C) (Guo et al., 2006). Thus, H_20_W_75_N_1_ increased root NR and protective enzyme, enhanced water-nutrient uptake and ultimately improved *g*_s_ which increased leaf gas exchange attributes and CO_2_ fixing prior to 82 DAE.

Interestingly, the H_20_W_75_N_1_ increased root physiological activity by 7.0–40.1% in 0–80 cm soil profile at 82 and 102 DAE. This increased water-N uptake and light absorption and utilization as well the activity of the reaction centers of PSII that contributed to photosynthate production (Reddy et al., 2004; Takahashi et al., 2013). Decreased root distribution under adequate water-nutrient application can decrease root dry matter and increase plant aerial parts dry matter accumulation of cotton crops (Ekmekçi et al., 2008). Available water-nutrient could promote accumulation of leaf dry matter which contributes to higher photosynthesis (Ekmekçi et al., 2008; Yang et al., 2011b). Water-nutrient application could increase available water-nutrient in the soil (Pettigrew, 2004), and enhance transport of assimilates from source to sink, which promote dry matter accumulation in reproductive structures (Radin et al., 1992; Pettigrew, 2004). Our data showed that H_20_W_75_N_1_ enhanced the biomass accumulation and allocation into reproductive organs, but decreased partitioning to roots.

## Conclusion

The presents study demonstrated that irrigation and N-fertilization managements significantly improved cotton plants morphological and physiological attributes under different water depths. H_20_W_75_N_1_ enhanced root physiological activity and simultaneously promoted the capacity of light capture (photosynthetic pigment) and conversion efficiency of photochemical energy, the utilization of light energy before full boll stage, and maintained leaf gas exchange parameters by relieving the adverse effects on roots after full boll stage. This improved dry mass accumulation in aerial parts and allocation into reproductive organs. Root physiological activity was positively correlated with chlorophyll, *F*_v_*/F*_m_, Y(II), and *g*_s_. Therefore, use of shallow irrigation with moderate water and nitrogen application is an effective strategy for optimal biomass accumulation and cotton yield.

## Author Contributions

HL initiated and designed the experiment. XT performed the experiments and collected the data. ZC analyzed the data and wrote the manuscript. DT, AK, and HL revised the manuscript.

## Conflict of Interest Statement

The authors declare that the research was conducted in the absence of any commercial or financial relationships that could be construed as a potential conflict of interest.

## Abbreviations

CAT: catalase
Chl *a* and *b*: chlorophyll *a* and *b*
*C*_i_: intercellular CO_2_ concentration
DAE: days after emergence
ETR(II): electron transport rate of PSII
*F*_v_*/F*_m_: potential quantum yield of PSII
*g*_s_: stomatal conductance
MDA: malondialdehyde
NR: nitrate reductase activity
*P*_n_: net photosynthetic rate
POD: peroxidase
Pro: proline
PS II: photosystem II
qN: non-photochemical quenching coefficient
qP: photochemical quenching coefficient
RLD: root length density
RMD: root mass density
RSD: root surface area density
SOD: superoxide dismutase
Y(II): PSII quantum yield in the light
